# Investigation of autistic traits through strategic decision-making in games with adaptive agents

**DOI:** 10.1038/s41598-017-05933-6

**Published:** 2017-07-17

**Authors:** Alexis B. Craig, Emily Grossman, Jeffrey L. Krichmar

**Affiliations:** 1 Department of Cognitive Sciences, University of California, Irvine, 2201 Social & Behavioral Sciences Gateway Building, Irvine, CA 92697 USA; 2 Department of Computer Science, University of California, Irvine, 3019 Donald Bren Hall, Irvine, CA 92697 USA

## Abstract

Autism Spectrum Disorders are characterized by difficulties in communicating and cooperating with other people. Impairment in Theory of Mind (ToM), the ability to infer what another person is thinking, may contribute to these social deficits. The present study assesses the relationship between autistic traits and decision-making in a socioeconomic game environment that measures ToM and cooperation. We quantified participant strategy during game play with computer agents that simulated aspects of ToM or fixed strategy agents with static behaviors or heuristics. Individuals with higher Autism Quotient (AQ) scores cooperated less than subjects with low AQ scores with the ToM agents. In contrast, subjects with higher AQ scores cooperated more with fixed strategy agents. Additionally, subjects with higher AQ scores spent more time than low AQ subjects signaling cooperative intent in games with fixed strategy agents while spending less time signaling cooperation with adaptive agents, indicating a preference toward systemizing behaviors in the face of uncertainty. We conclude that individuals with high levels of autistic traits are less likely to utilize ToM as a cognitive strategy, even when it is beneficial, to achieve a desired outcome.

## Introduction

Autism spectrum disorders (ASD) are typically characterized by difficulties in communicating and forming relationships with other people^1^. Beginning with the shift away from discrete diagnoses to the umbrella term featured in the DSM-V, the definition of autism has been formally changed to encompass a wider variety of disorders of social impairment including Asperger’s, childhood disintegrative disorder, and pervasive development disorder^2^.

Social deficits in individuals with ASD are in part attributed to impaired theory of mind (ToM), the ability to infer what another person is thinking, feeling, or perceiving^3^. The behavior of many individuals with high levels of autistic traits can be described as “systemizing,” meaning that such individuals tend to analyze systems by rules and patterns. Individuals with high levels of autistic traits often empathize less; that is, they tend not to predict and respond to the emotional state of others^4^. Furthermore, it has been shown that individuals with ASD have mentalizing deficits, the ability to imagine about oneself or others^5, 6^.

Common assessments of ToM abilities include tasks in which individuals must read stories and infer mental states of the characters (e.g., the Faux-Pas test and the Strange Stories test^5, 7^). While these tasks are successful in revealing positive correlations between utilization of ToM and the extent of autistic affectedness, narrative-based approaches rely on verbal ability and are abstracted by fiction. The field would benefit from the introduction of dynamic tasks that probe critical thinking and ToM in real time, and that can be conducted non-verbally to engage individuals with potentially limited verbal skills in social situations that minimize direct social contact.

The Stag Hunt is a socio-economic game in which cooperating and understanding the other player’s intent, both hallmarks of ToM, yields more beneficial outcomes. In this two-player game, each player must decide whether to cooperatively hunt a stag for a high payoff, or defect to hunt alone for a low payoff hare. The cooperative option derives higher reward, but comes at the risk of gaining no payoff in the event that the other player chooses hare. This game is commonly utilized in game theory because its payoff matrix encourages cooperation, a strategy that necessitates inference of the other player’s goals and the formation of trust between the players^8^. The Stag Hunt has also been adapted into a spatiotemporal variant in order to observe more complex interactions between players during their attempts to form a cooperative relationship over time^9^. Players move avatars around a game board, visually signaling their intention to hunt either a stag or hare by the path and position their avatar takes on the game board. This adaptation, played through repeated trials with the same player, allows for more complex strategizing and a deeper understanding of how one’s own actions can affect the other player, and vice versa.

In the present study, we investigate the relationship between autistic traits in typical individuals and implicit ToM during socioeconomic game play. Subjects played the repeated spatiotemporal Stag Hunt game with five different agents ranging from simple fixed strategies to adaptive strategies that simulated aspects of ToM. We quantified the level of autistic traits in our subclinical population using AQ scores from self-report survey responses^10^. The AQ test is a 50 question Likert-based survey querying the presence or absence of various behaviors typically associated with ASD.

We hypothesized that subjects with high levels of autistic traits would engage in less cooperative strategies as compared to subjects with low levels of autistic traits. This disinclination to engage implicit ToM should be most apparent while playing with an adaptive agent using aspects of ToM, in which signaling their intent can induce the agent to cooperate. In addition, we hypothesized that subjects with high levels of autistic traits would have difficulty inferring the intention of an agent with ToM attributes, which may be exhibited through differences in visual displays of intention during game play.

## Methods

### Human Participants

113 subjects (ages 18–33) were recruited in four separate sessions through the Experimental Social Science Laboratory (ESSL) at the University of California, Irvine (Table 1). Similar to previous reports^10^, there was little difference in AQ scores comparing males with females. The ESSL maintains contact information for a large population of undergraduate and graduate students that have agreed to participate in social and economic studies in exchange for monetary compensation. Subjects were not screened for characteristics (e.g. ethnicity, gender, age) beyond their current student status. Nine subjects were removed for failure to complete the AQ survey or the experimental task with full comprehension; the remaining 104 are included in the following analysis.

**Table 1:** Subject Characteristics. Mean (standard deviation).

Gender	Age	AQ
Female (n = 55)	20.2 (1.3)	20.0 (5.7)
Male (n = 49)	21.0 (2.2)	19.8 (5.2)
Overall (n = 104)	20.6 (1.8)	19.9 (5.4)

The experimental protocol was reviewed and approved by the UCI Institutional Review Board, and informed consent was obtained from all subjects. The present study had full ethical approval from the University of California, Irvine Institutional Review Board (IRB) and was carried out in accordance with the IRB’s relevant guidelines and regulations.

### Stag Hunt Software

**Figure 1:**
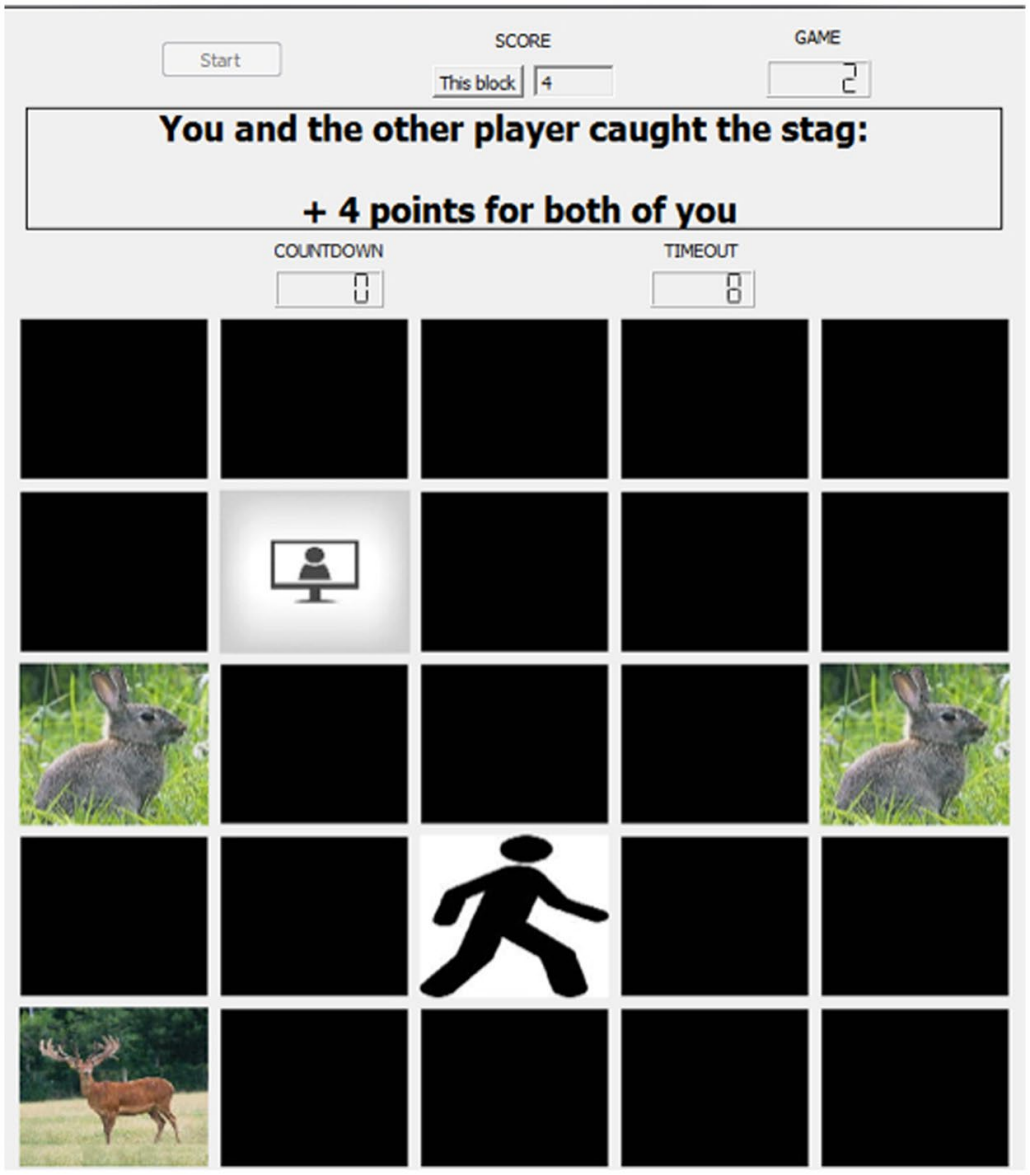
Stag Hunt game environment. Subjects moved their avatar (stick figure) across the 5 × 5 game board using the arrow keys in order to catch a low payoff hare individually, or attempt to catch a high payoff stag with the computer agent (silhouette in computer screen). To catch a stag, both the subject and the agent must move their avatars into a square directly adjacent to the stag square, and the subject must press the Space bar. To catch a hare, the subject must move their avatar on top of a hare square and press the Space bar. When a prey was successfully caught by one of the subjects, the game ended and points were awarded based on prey.

In Craig *et al*.^9^, software was developed to create a spatiotemporal version of the Stag Hunt. The game board (Fig. 1) included a 5 × 5 grid of squares on which a human player and computer agent each controlled an avatar. The game board included hare squares positioned at the leftmost and rightmost squares of the middle row of the board, and a stag image placed on a random square at the beginning of each game. Two hares were included in order to prevent one player from having a significant location advantage derived from the random placement. The player avatars started on a random square on the game board such that they did not appear directly next to a hare, a stag, or each other.

The human player moved their avatar using the left, right, up, and down arrows. Diagonal movement was not allowed for either player. The human player was able to move as often as they liked, while the agent moved at randomized intervals between 300 and 900 ms, timed to reduce predictability and to approximate the range of reaction times exhibited by humans.

Pilot testing was conducted with subjects who were not part of the study to test the agents and set open parameters, such as the reaction time of humans. Pilot testing was also important for determining learning rates and the number of games per block. This ensured that the adaptive agents had sufficient time for learning to stabilize and for the agents to demonstrate tendencies based on the subject’s behavior. In addition to subject pilot testing, we ran simulations in which the agents played each other. These simulations were used to verify that the different agents responded appropriately, and to determine the expected number of prey caught during a block of games.

A successful hare capture required the player to be on the same square as the hare and to press the space bar, which awarded one point to the first player who initiated a capture. A successful stag capture was worth 4 points to each player and required both players to be on squares adjacent to the stag square. Successfully capturing the hare or stag ended the game. If neither the stag nor the hare was captured within ten seconds, the game timed out and no players received points.

### Computer Agents

Participants played the Stag Hunt against an agent with a fixed strategy and an agent that simulated aspects of ToM and planning, the ToMPlan agent. The fixed strategy agent, which we call Random, was randomly assigned a prey (stag or hare) at the onset of each game and moved directly toward that goal without deviation. For each agent, subjects played multiple successive games (blocks), allowing the agent to develop game outcome-based contingent strategies.

The ToMPlan agent simulated two primary mechanisms influencing human decision-making: planning and theory of mind. It was predictable in that its behavior was increasingly influenced by experience during a game, but an element of unpredictability was implemented by using a Softmax function. The ToMPlan agent used a variation of an actor-critic model^11^, which was based on our previous work^9^. The actor-critic model was comprised of state tables associating all possible configurations of the game board with their expected value under hare or stag hunting. Similar to Craig *et al*.^9^, the model updated state tables for a Reward Critic, Cost Critic, and Actor. These tables were initialized to zero and the learning rate for the actor-critic model was set to 0.10. Each state was designated by: (1) the player’s distance from hare, (2) the agent’s distance from hare, (3) the player’s distance from stag, and (4) the agent’s distance from stag. The distances were calculated using Euclidean distance and then truncated to the nearest integer value. Although, a Manhattan distance could also have been used, the assumption was the players would perceive distances to targets despite not being able to move diagonally. Given that players were allowed to move in real time rather than waiting between each move, a quick left and up move would suffice to make a diagonal move. Using Euclidean distance, as opposed to Manhattan distance, also reduced the state space, which stabilized learning. Player tokens could be, at most, a distance of five from its current position to a square adjacent to the stag, and a distance of, at most, four from its current position to the square of the nearest hare. Agents were allowed to move on top of hares, but not stags. Therefore, each state table for the game board had 400 possible states (4 × 4 × 5 × 5). The state table values were based on the agent’s experience and were updated in real-time after either the subject or the agent moved.

The Reward Critic state table contained a weight that corresponded to the expected reward at the current state. Reward was defined as the payoff received at the end of a game as given by the payoff matrix (i.e., one point for hare capture, four points for stag capture). Similarly, the Cost Critic state table contained a weight that corresponded to the expected cost at the current state. Cost was defined as the perceived loss on a hunt. For example, if the agent was hunting a stag and the human caught a hare, the cost would be −4. The Actor state table contained two weights for each state: one for the likelihood to hunt hare and the other for the likelihood to hunt stag in a given state. Before every move by an agent in a game, the Actor used the expected value for hunting hare or stag at the current state to generate a choice probability using a Softmax function (β = 1). The agent moved one square closer to the selected target. For more on the actor-critic model, see Craig *et al*.^9^.

**Figure 2:**
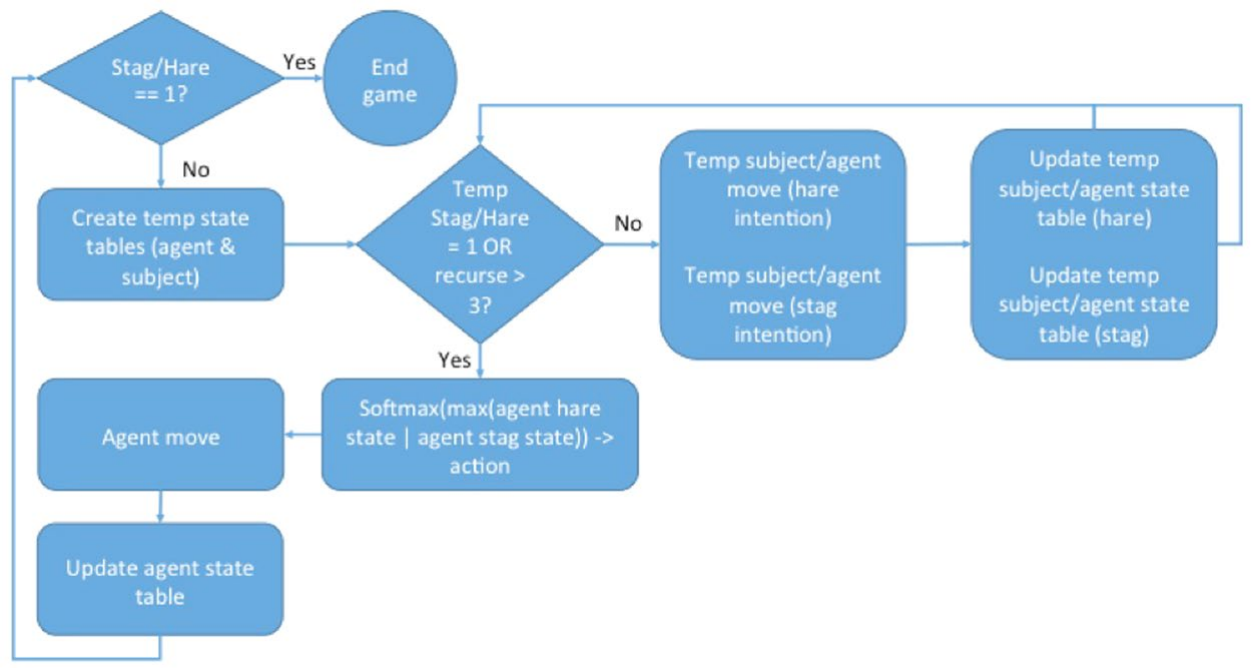
Flow chart of the theory of mind planning (ToMPlan) model. On each turn, if an endgame was not reached through a stag or hare capture, the model would decide upon an action of either moving closer to the hare or the stag using state table information run through a Softmax function. The agent would then move one square on the game board, update the state tables, and iterate again. While an endgame state was not reached, a temporary copy of the state tables was made and a sample game for both hare and stag outcome was simulated until either endgame state was reached or three levels of recursion were completed. The prey with the simulated game containing the highest valued end state was selected, and this state was run through the Softmax function to return a prey to act upon. This model held state tables for both the agent and the subject.

The ToMPlan agent extended the basic actor-critic model to mimic mental simulation and planning (Fig. 2). It maintained real-time state tables of both the agent’s and the subject’s expected values for stag and hare hunting for each board configuration. The asynchronous timing of moves was not an issue, since the time intervals of the agent moves were similar to that of the subjects, and the number of moves by each player per game was comparable. State tables were updated after each agent’s move and after each subject’s move. State tables were also used to simulate a full game prior to making each move in order to select the prey with perceived higher profitability based on the simulation’s outcome. The agent maintained a separate set of tables (i.e., a projected state table of the subject and the agent) that were updated after the subject’s moves in order to simulate the subject’s knowledge. This agent was able to learn in real time, using both its own and the other player’s behavior, plan ahead based on projected game outcomes, and form a conception of the other player’s mental state based on their payoff history. In addition to being used to update the in-game weights after a move on the game board, the agent “mentally” simulated a full game using the player’s state tables to approximate the player’s likely strategy based on the current setup of the board. To model planning, two “mental” simulations were conducted by simulating game play for hunting a stag or a hare. Given the fast pace of the game, we assumed that the mental simulation could only recursively plan three moves ahead (e.g., if I move here, then you would move there) or less in the case that a prey was caught. Bounding the recursion at three moves also prevented computational explosion that might cause the agent not to respond in real-time. A copy of the state tables was made to temporarily update during this simulated game (Temp in Fig. 2). The prey in the mental simulation that ended in the highest Actor state value from the temporary Actor state table was selected to pursue for the agent’s next move.

Four other agents were tested. An Actor-Critic agent used the Stag Hunt rewards and costs to develop policies, but did not have mental simulation. Subjects playing with the Actor-Critic agent showed similar effects compared to play with ToMPlan, but of a lesser magnitude. We considered a Win-Stay-Lose-Shift agent, which kept its target if it won the previous game and switched if it lost. We also considered a Plan agent, which used the same planning mechanism as the ToMPlan agent but did not maintain a state table that considered the other player’s position on the board. Neither the WSLS agent nor the Plan agent evoked strong differential behavior in subjects, because subjects learned to always hunt stags. For these reasons, we report only on the two most polarizing agents, the agent that made a random prey choice at the beginning of the game (Random) and the agent that had aspects of Theory of Mind and planning (ToMPlan).

### Experimental Design

Subjects read written instructions outlining how to play the Stag Hunt, then played a 15-game training block against the Random agent. Following the practice, subjects participated in five blocks against different agents (in randomized order), with 50 games per block. Subjects were allowed to complete the task at their own pace. Between each block, the subjects filled out a written survey querying their own behavior and the perceived cooperation of the agent they were playing with.

After finishing the experiment, subjects completed a written demographic survey collecting personal information regarding their age, major, experience with video games, whether English was their first language, and whether they were willing to be contacted for future research opportunities. They then completed the 50-question Autism Spectrum Quotient (AQ) test for adults (University of Cambridge’s Autism Research Centre). Subjects were compensated US$7 plus a variable US$0-$15 of performance earnings that corresponded to their score during a randomly selected block of the computer experiment. Each point was worth 7.5 cents, and the total payment was rounded to the closest dollar.

### Analysis

Unless otherwise specified, all proceeding analyses were performed in MATLAB (Mathworks, Inc.) and tested for significance using a two-sample Kolmogorov-Smirnov hypothesis test. Each significance threshold was corrected for error of p < 0.05 using Bonferroni correction.

Cooperation and intent were measured for subject play with each agent. The primary metric of cooperation used in our analysis of the Stag Hunt was the number of successful stag captures per block. In a given block, there was the opportunity to catch 50 stags. Subject intention signaling was measured by path deviation and loitering metrics. Path deviation was calculated by finding the length of the direct path (distance between the first and last moves for each game of each subject) and subtracting that number from the subject’s total distance traveled in each game (calculated by summing the distances between each move). Loitering was measured in seconds from the moment the player reached a square adjacent to the stag until either the player moved to a non-adjacent square, or the end of the game was reached.

The state tables produced by the adaptive agents contain detailed information regarding target choice preferences in relation to game board configuration. State tables were analyzed to calculate the probability of hunting stags and hares under different board configuration states.

## Results

Scores on the AQ test across the subject population ranged from 9 to 36 points (out of 50), with greater scores indicating higher affectedness by autistic traits. Subjects differed in their behavior and in their responses to different agents depending on their level of autistic traits as measured by AQ. Overall, subjects with high AQ scores tended to cooperate less and had trouble discerning the intention of adaptive agents.

### Cooperation

**Figure 3:**
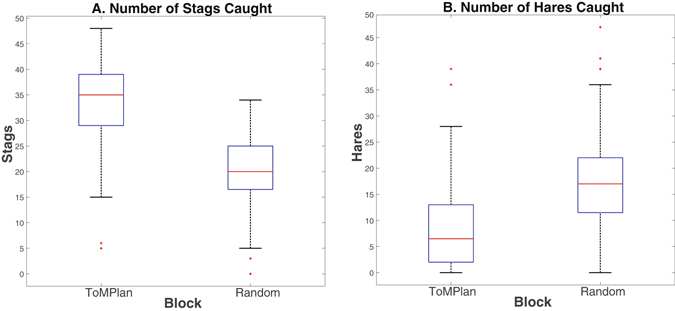
Hunting behavior by subjects playing the Stag Hunt. (**A**) Number of stags caught over all games by block. Subjects caught significantly more stags ToMPlan condition (p < 0.001). (**B**) Number of hares caught by subjects over all games by block. Subjects caught significantly more hares in the Random condition (p < 0.001). In the graphs, the x-axis is blocked by agent type, while the y-axis is total stags caught during block (**A)** and total hares caught by subject during block, out of a maximum of 50 (**B**).

Analysis of cooperative behavior (i.e., hunting stags) indicated that subjects were sensitive to the differences between the simulated agents and changed their play accordingly. Subjects caught significantly more stags in the ToMPlan condition (p < 0.001; Fig. 3A), and significantly more hares in the Random condition overall (p < 0.001; Fig. 3B).

**Figure 4:**
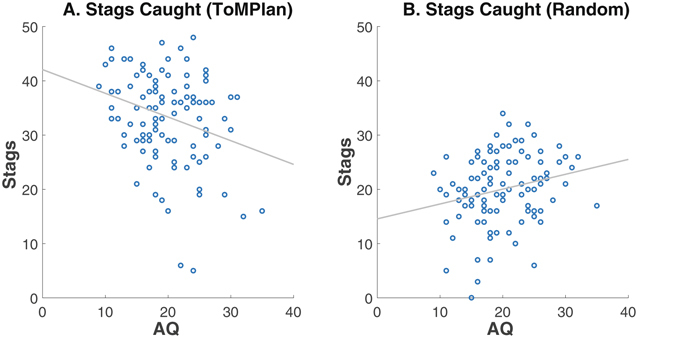
The number of stags caught as a function of AQ. (**A**) ToMPlan. The number of stags caught in the ToMPlan condition was significantly negatively correlated with AQ (r = −0.276, p < 0.002). (**B**) Random. The number of stags caught in the Random condition was significantly positively correlated with AQ (r = 0.234, p < 0.008). The x-axis is AQ ranging from 0–40, the y-axis is the number of stags out of a possible 50, and the regression line is included.

The relationship between AQ and cooperation revealed distinct patterns depending on agent type. Cooperation, as measured by stag captures, was negatively correlated with AQ in the ToMPlan condition (r = −0.276, p < 0.002), and positively correlated with AQ in the Random condition (r = 0.234, p < 0.008; Fig. 4). Low AQ subjects captured more stags than high AQ subjects when playing with the adaptive agent, whereas high AQ subjects captured more stags than low AQ subjects in the Random condition. The implication is that subjects with high AQ did not recognize and develop strategies for eliciting cooperative behavior from the ToMPlan agent, evidence for difficulty understanding the adaptive agents’ intention.

This does not imply, however, that high AQ subjects did not understand the purpose and goals of the Stag Hunt game. High AQ subjects could cooperate with the simple Random agent, and were even more successful at capturing stags than lower AQ individuals. In this condition, being predisposed to systemizing strategies rather than trying to understand another player’s intention resulted in better scores.

**Figure 5:**
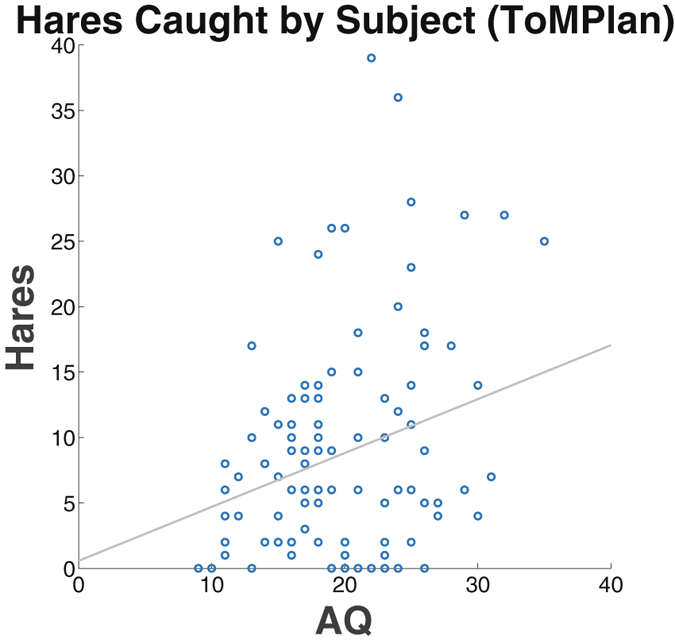
Average number of hares caught by subjects as a function of AQ during the ToMPlan condition. A significant positive correlation was found in the ToMPlan condition (r = 0.264, p < 0.003), indicating that subjects with higher AQ tended to catch more hares than in lower AQ subjects. The x-axis is AQ ranging from 0–40, the y-axis is the number of hares out of a possible 50, and the regression line is included.

Hare hunting is typically preferential when cooperation cannot be achieved. In support of the finding that subjects with High AQ tended to not cooperate (i.e., stag hunting) when playing an agent with ToM attributes, there was a positive correlation between AQ and the number of hares caught during game play with the ToMPlan agent (see Fig. 5; r = 0.264, p < 0.003). High defection rates (i.e., hare hunting) may suggest that high AQ subjects could not interpret the intent of ToMPlan agent^12^, or the possibility that they preferred to systemize rather than mentalize. Furthermore, the tight time constraints of the Stag Hunt game may have played a role in the tendency to utilize a systematic strategy^13^.

Together, these findings support the hypothesis that high AQ subjects were less likely to engage in implicit ToM, reminiscent of impairment characteristic of clinical ASD subjects^6^. Individuals with high AQ were less cooperative when faced with a player that had the ability to alter its decisions depending on changing context. In other words, they had trouble discerning the intentions of the agent, which simulated attributes of ToM. Alternatively, these results may suggest that high AQ subjects utilized a more systematic strategy that worked well with the Random agent, but not with the ToMPlan agent, especially under the tight time constraints of the Stag Hunt game.

### Intent

**Figure 6:**
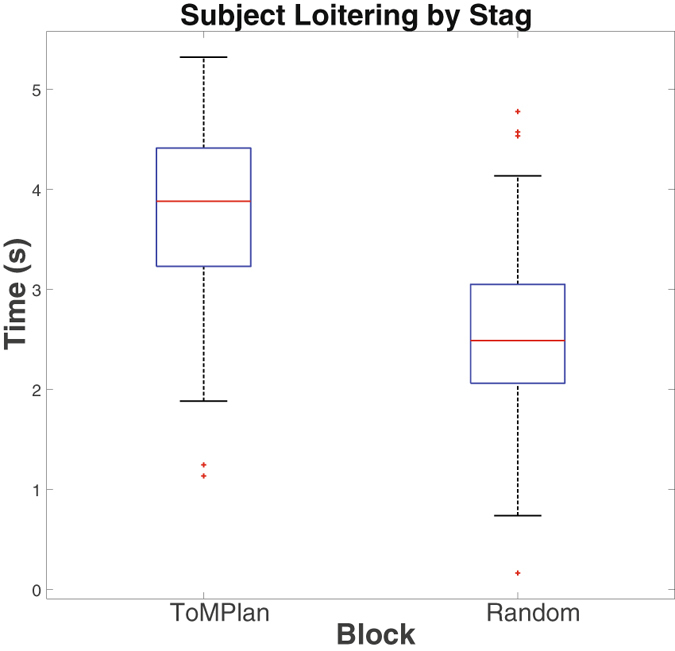
Average time in seconds spent “loitering” next to a stag during a block. Loitering was significantly less for the Random condition (p < 0.001). The x-axis is blocks by agent type, while the y-axis is time in seconds.

A clear signal of intent to cooperate would be to position oneself adjacent to the stag and wait for the other player, which we define as loitering. In general, subjects loitered significantly less when playing with a Random agent than a ToMPlan agent (p < 0.001; Fig. 6). This suggests that subjects thought the ToMPlan could understand intentions. In addition, we measured path deviation in which a subject might indicate intent (see Table 2). However, we saw no significant difference between the Random and ToMPlan conditions, and no effect of AQ.

**Table 2:** Behavioral metrics for subjects by mean and standard deviation.

Metric	ToMPlan	Random	Effect Size ToMPlan vs. Random
Stags caught	33.365 (8.590)	20.019 (6.348)	0.662
Hares caught	8.788 (8.487)	17.269 (9.371)	−0.429
Subject path dev.	0.412 (0.342)	0.377 (0.340)	0.051
Subject loiter	3.754 (0.871)	2.551 (0.830)	0.577

**Figure 7:**
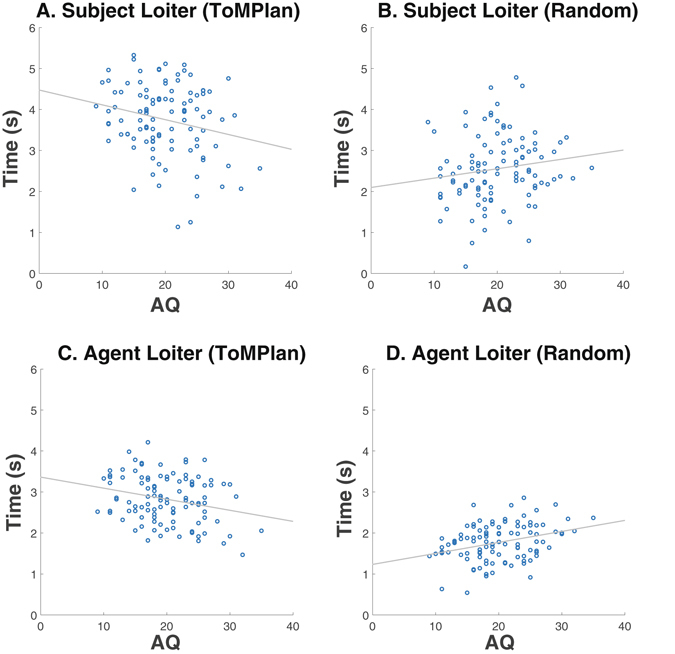
Average time in seconds spent “loitering” as a function of AQ. (**A**) Subject loitering when playing with a ToMPLan agent was significantly correlated with AQ (r = −0.225, p < 0.01). (**B**) Subject loitering when playing with a Random agent was not significantly correlated with AQ, but showed a positive trend. (**C**) ToMPlan agent loitering was significantly correlated with AQ (r = −0.255, p < 0.004). (**D**) Random agent loitering was significantly correlated with AQ (r = 0.326, p < 0.001). The x-axis is AQ ranging from 0–40, the y-axis is the average amount of time in seconds the player spent next to a stag per game, and the regression line is included.

We measured the relationship between AQ and the extent to which subjects or agents engaged in the signaling of their intentions by loitering next to a stag during game play. The amount of time both subjects and agents spent loitering next to a stag in the ToMPlan condition was negatively correlated with AQ when examining subject loitering (Fig. 7A: r = −0.225, p < 0.01) and agent loitering (Fig. 7C: r = −0.255, p < 0.004). The amount of time the Random agent spent loitering next to a stag was positively correlated with AQ (Fig. 7D, r = 0.326, p < 0.001). The amount of loitering by subjects when playing with the Random agent showed this positive trend, but it did not reach significance (Fig. 7B).

These effects provide further evidence that subjects with higher AQ tend to spend less time signaling intent in the ToMPlan condition. These trends are also indicative of increased systemizing behavior in high AQ subjects, as these subjects allowed the Random agent more time to signal its intention to catch stags. Because the Random agent’s strategy was predictable within game by always taking a direct path toward its target, subjects with high systemizing tendencies could pick up on the pattern and use it to their advantage. The ToMPlan agent on the other hand was unpredictable because of its constantly adapting behavior, making it more difficult to recognize systematic patterns. Systemizing behavior, in which individuals tend to analyze systems by rules and patterns, has been observed in ASD^14–16^.

### Agent state tables

**Figure 8:**
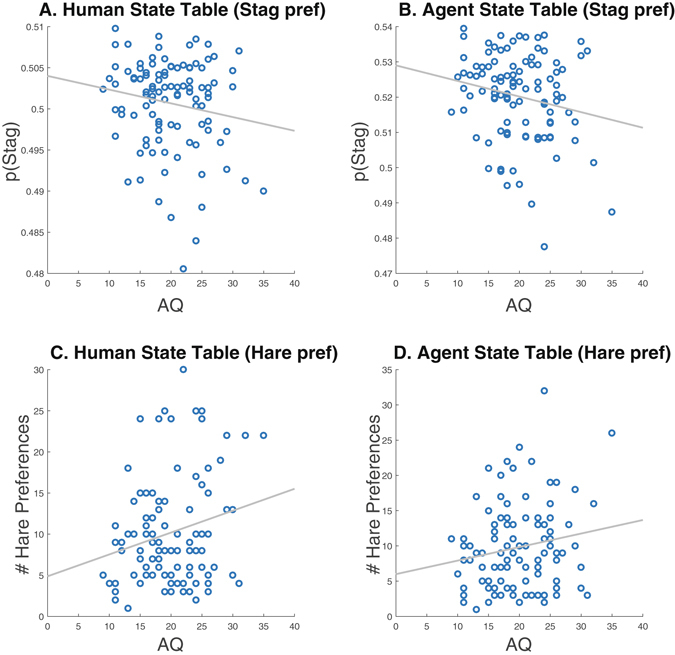
Target preference as measured by the human and agent actor state tables constructed by the ToMPlan agent. (**A**) Average weights for stag preference in the human state table. (**B**) Average weights for stag preference in the agent state table. Subject AQ was negatively correlated with state table weights for the agent (r = −0.195, p < 0.05). (**C**) Number of entries in the human state table, where the likelihood to hunt hare was greater than 60%. AQ was positively correlated with the likelihood to hunt hare (r = 0.224, p < 0.022). (**D**) Number of entries in the agent state table, where the likelihood to hunt hare was greater than 60%.

The consequence of neither engaging in cooperative behavior nor signaling intent to do so is reflected in state tables created by the ToMPlan agent. Based on its experience, the ToMPlan agent constructs tables to choose an action based on the game board state. The ToMPlan agent also constructs tables to predict the human player’s action based on the game board state. By analyzing these state tables, we were able to identify trends that underscore the findings above (Fig. 8). Subject AQ was negatively correlated with state table weights for the agent indicative of the agent’s propensity to hunt stags when playing with low AQ subjects (r = −0.195, p < 0.05; Fig. 8B). The same trend was found in the subject state tables, although this did not reach significance (r = −0.16, p < 0.1; Fig. 8A). Likewise, AQ was positively correlated with the number of states in the subject state table where the likelihood to hunt hare was greater than 60% (r = 0.224, p < 0.022; Fig. 8C). The same trend was found in the agent state tables, although this did not reach significance (r − 0.17, p < 0.08; Fig. 8D). This indicates that subjects with higher AQ showed they were less amenable to stag hunting in a way that was apparent to the ToMPlan agent.

## Discussion

By using sophisticated computer agents in a socioeconomic game, known as the Stag Hunt, we investigated the relationship between theory of mind and autistic traits. When playing with an agent that had aspects of ToM, subjects with higher AQ scores had difficulty: (1) signaling intent, (2) understanding intent, and (3) cooperating with other agents. However, this was not the case when high AQ subjects played against an agent with a fixed strategy. These results suggest that high AQ subjects may have utilized different strategies such as systemizing behavior and tending to not consider another’s intent during cognitive tasks.

Subjects with higher incidence of autistic traits were less likely to engage in cooperative behaviors when playing games with the ToMPlan agent. AQ score was negatively correlated with the amount of cooperation when playing with an agent that simulated ToM, and AQ score positively correlated to the amount of cooperation when playing with a simplistic, fixed strategy agent that randomly chose a prey at the start of a game. This suggests that subjects with high levels of autistic traits may have had trouble understanding the intent of an agent that simulates ToM, much like individuals with clinical ASD^17, 18^.

Individuals with high AQ scores were more successful hunting stags with Random agents than low AQ individuals. This implies that higher AQ subjects had an easier time understanding another player with fixed strategies, or that a systemizing strategy, which high AQ subjects might favor, was beneficial under these conditions. This is further supported by the positive correlation between time spent loitering next to the stag and AQ when playing with the Random agent.

High AQ subjects did not, however, loiter when playing with the ToMPlan agent. The state tables reveal that the subject’s behavior shaped the agent’s actions towards hare hunting in games with high AQ subjects. That is, the ToMPlan agent learned that the subjects with high AQ are less likely to hunt stags, and adjusted its behavior accordingly. The implication is that high AQ subjects have more difficulty understanding the behavior of adaptive agents having aspects of ToM, which likely stems from implicit ToM impairment^5^.

Taken together, these results demonstrate that a non-verbal task, such as the Stag-Hunt, can induce differential behavior among subjects whose autistic traits differ, and that these differences may be related to variations in implicit theory of mind, especially when a sophisticated player challenges subjects.

### Cooperation

In research closely related to the AQ test, the Empathizing/Systemizing Quotient tests are also used to assess the degree of autistic traits in general population individuals^14, 16^. These studies have shown that individuals with autism and high levels of autistic traits tend to score high on the systemizing scale and low on the empathizing scale, meaning that such individuals tend to analyze systems by rules and patterns, rather than predicting and responding to mental states and emotions^4^. The finding that high AQ subjects were more successful in catching stags when playing the Random agent indicates a strategy consistent with a high systemizing mentality, and may be a good strategy for analyzing the path pattern of this fixed strategy agent.

The low AQ subjects, on the other hand, were less likely to capitalize on stag hunting opportunities presented by the Random agent. This may be because the low AQ subjects tried to decipher and influence the intentions of this fixed strategy agent, which was not possible. Alternatively, the low AQ subjects may have selected a strategy of planned defection. Previous studies in game theory have shown that emotion plays an important role in decision-making during social games^19^. The phenomenon of acting against one’s best interests in order to seek revenge on a party that one believes has wronged them is well established in game theory, especially in tasks such as the Ultimatum Game^20^. It is possible that low AQ subjects, after several failed attempts at cooperation, became upset with the agent and acted out against them by defecting. As empathy and emotion are interlinked with ToM processing^21^, the difference between high and low AQ subjects likely revolve around the recruitment of ToM in decision-making.

### Additions to Prior Research

ToM responses can be evoked in two different ways: explicitly and implicitly. Most commonly, ToM is probed through narrative tasks that *explicitly* require the participant to imagine what another person is thinking. One common example, the False Belief task, tests a subject’s ability to represent another person’s knowledge separately from their own, allowing for the fact that other people may not know what you know. The False Belief task is trivial for typical subjects, yet young children and some individuals with ToM impairments are not able to pass it^5, 22^. While less commonly utilized, ToM can also be investigated through non-narrative tasks that provide their context *implicitly* through an animation or a game environment. Through clips of animated shapes using biological or random motion, Castelli *et al*. was able to probe ToM in both neurotypical and cognitively able subjects with autism, identifying differences in brain activity within the ToM network^23^. The addition of eye-tracking hardware to ToM studies can also be a useful probe into cues that signal ToM, especially in studies that incorporate individuals with autism because attempts to engage in ToM can be interpreted through eye gaze. For instance, individuals with autism are more likely to look at the mouth and less likely to look at the eyes of a social partner, indicating that the cues they are using are different from neurotypical individuals^24^. The concept of explicit vs. implicit ToM tasks is a key component in using ToM tasks to study autism, as it has been theorized that the atypical activity of the ToM network in the brain differs depending on which type of task is employed^6^. It is especially important to investigate implicit ToM, as the process arises spontaneously and is therefore more difficult to accurately assess.

The present study adds to the comparatively small field of implicit ToM research, creating a non-verbal paradigm to evoke implicit ToM through cooperation. We have shown through metrics of cooperation and intention signaling that autistic traits correlate to differential behavior in this task, likely with the difference residing in ToM impairment.

In an effort to simulate the complex strategies of a player, Yoshida *et al*. had subjects play the spatiotemporal Stag Hunt with a model-based computer agent, which used fluctuating levels of ToM sophistication defined by the level of recursion of thinking about the other person’s mental state^25, 26^. In a follow up experiment, Yoshida *et al*. showed that high functioning clinical ASD subjects tended to use a fixed strategy, selecting the same prey for a majority of games without much switching, whereas neurotypical subjects tended to behave more flexibly^27^. ASD subjects had difficulty interpreting the intentions of agents exhibiting higher levels of ToM sophistication. These agents lacked the ability to react in real-time, instead switching their level of sophistication at random. This behavior does not lend itself well to the assessment of social interaction, as the other player would not reliably behave in a manner that appeared to be influenced by the subject.

The present study goes beyond Yoshida’s work in that the agents used in the present experiment adapt in real-time rather than switching between fixed models at a designated interval. The adaptive agent entered the Stag Hunt environment naïve and developed a strategy over repeated games based on both the behavior of the other player and the outcomes of the games.

However, the present study does have its limitations. Firstly, we did not directly measure systemizing, although the results suggest high AQ subjects were applying rules. Secondly, it is difficult to discern whether subjects were ascribing ToM to the ToMPlan agent, or whether they had a ToM deficit. Subject questionnaires were inconclusive and future studies will need some way to directly measure this, such as a questionnaire specifically for ToM, or a complementary ToM task. Lastly, although the AQ test has been used to show autistic traits, it is usually used in conjunction with neurotypical subjects. It would be of interest to have subjects diagnosed with ASD play the Stag Hunt with the agents described here. This is something our group may do in the future, or would be willing to make the game software available to a group having access to such a subject pool.

With the present study, we were able to show that differences in autistic traits, as measured by AQ, can highlight differences in ToM usage when a sophisticated agent with ToM attributes challenges subjects. This novel approach could be used to probe implicit theory of mind in the general population and in clinical populations.
